# Supplementary material for: Modeling glioblastoma heterogeneity as a dynamic network of cell states
Source: Mol Syst Biol. 2021 Sep 16;17(9):e10105. doi: 10.15252/msb.202010105 (PMC8444284; doi:10.15252/msb.202010105)
Supplement: Supplementary file 5 — Source Data for Figure 3 [file MSB-17-e10105-s001.zip › Figure3A_sourcedata/GSEA_3065/nosigdb_state5.GseaPreranked.1622718435914/RICHARDS_ZHONG_NATURE_2018_OPC_UPREG.html]

Details for gene set RICHARDS\_ZHONG\_NATURE\_2018\_OPC\_UPREG[GSEA]

|  || Dataset | state5 |
| Phenotype | NoPhenotypeAvailable |
| Upregulated in class | na\_pos |
| GeneSet | RICHARDS\_ZHONG\_NATURE\_2018\_OPC\_UPREG |
| Enrichment Score (ES) | 0.579326 |
| Normalized Enrichment Score (NES) | 2.201174 |
| Nominal p-value | 0.0 |
| FDR q-value | 0.0 |
| FWER p-Value | 0.0 |
Table: GSEA Results Summary

  

Fig 1: Enrichment plot: RICHARDS\_ZHONG\_NATURE\_2018\_OPC\_UPREG      
 Profile of the Running ES Score & Positions of GeneSet Members on the Rank Ordered List

  

| PROBE | GENE SYMBOL | GENE\_TITLE | RANK IN GENE LIST | RANK METRIC SCORE | RUNNING ES | CORE ENRICHMENT || 1 | NOVA1 |  |  | 10 | 0.870 | 0.0354 | Yes |
| 2 | PTPRZ1 |  |  | 12 | 0.862 | 0.0714 | Yes |
| 3 | CD9 |  |  | 14 | 0.857 | 0.1072 | Yes |
| 4 | BCAN |  |  | 15 | 0.850 | 0.1429 | Yes |
| 5 | PTN |  |  | 19 | 0.824 | 0.1771 | Yes |
| 6 | CD63 |  |  | 24 | 0.769 | 0.2090 | Yes |
| 7 | SOX6 |  |  | 28 | 0.754 | 0.2403 | Yes |
| 8 | SCRG1 |  |  | 36 | 0.715 | 0.2694 | Yes |
| 9 | PLAT |  |  | 37 | 0.714 | 0.2994 | Yes |
| 10 | GPM6B |  |  | 41 | 0.702 | 0.3285 | Yes |
| 11 | B2M |  |  | 61 | 0.601 | 0.3515 | Yes |
| 12 | PON2 |  |  | 84 | 0.546 | 0.3720 | Yes |
| 13 | LRRC4C |  |  | 101 | 0.525 | 0.3922 | Yes |
| 14 | SEMA5A |  |  | 111 | 0.516 | 0.4129 | Yes |
| 15 | ITGB8 |  |  | 126 | 0.503 | 0.4324 | Yes |
| 16 | LSAMP |  |  | 132 | 0.497 | 0.4527 | Yes |
| 17 | TTYH1 |  |  | 141 | 0.489 | 0.4723 | Yes |
| 18 | SCD5 |  |  | 173 | 0.460 | 0.4882 | Yes |
| 19 | PDGFRA |  |  | 175 | 0.460 | 0.5073 | Yes |
| 20 | ALCAM |  |  | 225 | 0.422 | 0.5195 | Yes |
| 21 | SERPINE2 |  |  | 238 | 0.414 | 0.5355 | Yes |
| 22 | SPARC |  |  | 327 | 0.370 | 0.5412 | Yes |
| 23 | SEZ6L |  |  | 422 | 0.334 | 0.5446 | Yes |
| 24 | BCHE |  |  | 425 | 0.333 | 0.5584 | Yes |
| 25 | EDNRB |  |  | 519 | 0.306 | 0.5608 | Yes |
| 26 | ITM2C |  |  | 551 | 0.297 | 0.5698 | Yes |
| 27 | CADM2 |  |  | 739 | 0.253 | 0.5594 | Yes |
| 28 | OLIG1 |  |  | 809 | 0.239 | 0.5617 | Yes |
| 29 | LMF1 |  |  | 832 | 0.235 | 0.5691 | Yes |
| 30 | SPATA6 |  |  | 852 | 0.232 | 0.5767 | Yes |
| 31 | PDE4B |  |  | 968 | 0.215 | 0.5728 | Yes |
| 32 | NXPH1 |  |  | 990 | 0.212 | 0.5793 | Yes |
| 33 | SLC35F1 |  |  | 1092 | 0.199 | 0.5763 | No |
| 34 | ANGPTL2 |  |  | 1391 | 0.162 | 0.5497 | No |
| 35 | CST3 |  |  | 1602 | 0.141 | 0.5321 | No |
| 36 | CSPG5 |  |  | 1670 | 0.136 | 0.5302 | No |
| 37 | EPN2 |  |  | 1688 | 0.134 | 0.5340 | No |
| 38 | MMP2 |  |  | 1824 | 0.125 | 0.5241 | No |
| 39 | RFTN2 |  |  | 1867 | 0.121 | 0.5244 | No |
| 40 | CD82 |  |  | 2145 | 0.102 | 0.4976 | No |
| 41 | KANK1 |  |  | 2429 | 0.083 | 0.4694 | No |
| 42 | FIBIN |  |  | 2599 | 0.074 | 0.4535 | No |
| 43 | EPB41L2 |  |  | 2855 | 0.060 | 0.4274 | No |
| 44 | ETV1 |  |  | 2867 | 0.059 | 0.4287 | No |
| 45 | ETV5 |  |  | 2957 | 0.055 | 0.4210 | No |
| 46 | RAMP1 |  |  | 3221 | 0.040 | 0.3932 | No |
| 47 | ARC |  |  | 3455 | 0.029 | 0.3682 | No |
| 48 | CA10 |  |  | 3556 | 0.025 | 0.3581 | No |
| 49 | SIRT2 |  |  | 3589 | 0.024 | 0.3555 | No |
| 50 | SPRY4 |  |  | 3821 | 0.015 | 0.3302 | No |
| 51 | CNTN1 |  |  | 4200 | -0.001 | 0.2878 | No |
| 52 | SOX8 |  |  | 4306 | -0.006 | 0.2763 | No |
| 53 | C2orf80 |  |  | 4540 | -0.016 | 0.2508 | No |
| 54 | SPRY1 |  |  | 4844 | -0.027 | 0.2179 | No |
| 55 | GATM |  |  | 6035 | -0.076 | 0.0876 | No |
| 56 | LIMA1 |  |  | 6339 | -0.092 | 0.0575 | No |
| 57 | GNG7 |  |  | 6584 | -0.107 | 0.0346 | No |
| 58 | OLIG2 |  |  | 6595 | -0.108 | 0.0380 | No |
| 59 | TSC22D4 |  |  | 6599 | -0.108 | 0.0422 | No |
| 60 | ARL4A |  |  | 6930 | -0.131 | 0.0107 | No |
| 61 | DUSP6 |  |  | 7258 | -0.162 | -0.0192 | No |
| 62 | PLEKHB1 |  |  | 7506 | -0.191 | -0.0389 | No |
| 63 | PSAT1 |  |  | 7709 | -0.220 | -0.0523 | No |
| 64 | TRIB2 |  |  | 7741 | -0.224 | -0.0464 | No |
| 65 | PMP2 |  |  | 8058 | -0.288 | -0.0698 | No |
| 66 | MT3 |  |  | 8279 | -0.348 | -0.0799 | No |
| 67 | CNP |  |  | 8294 | -0.352 | -0.0667 | No |
| 68 | ANXA5 |  |  | 8626 | -0.510 | -0.0824 | No |
| 69 | RHOC |  |  | 8823 | -0.676 | -0.0761 | No |
| 70 | RAB31 |  |  | 8831 | -0.687 | -0.0480 | No |
| 71 | DBI |  |  | 8895 | -0.764 | -0.0230 | No |
| 72 | S100B |  |  | 8908 | -0.784 | 0.0085 | No |
Table: GSEA details [plain text format]

  

Fig 2: RICHARDS\_ZHONG\_NATURE\_2018\_OPC\_UPREG: Random ES distribution      
 Gene set null distribution of ES for **RICHARDS\_ZHONG\_NATURE\_2018\_OPC\_UPREG**
